# A novel GLRB mutation in neonatal hyperekplexia with divergent EEG findings: a case series

**DOI:** 10.1097/MS9.0000000000003258

**Published:** 2025-04-04

**Authors:** Motaz Tamimi, Majd Oweidat, Hamza Atawneh, Mohammed Alra’e, Bahaa Amr, Mohammad Gharaibiah, Mohammed Aldwaik, Mariam Alqam

**Affiliations:** a College of Medicine, Hebron University, Hebron, West Bank, Palestine; b Division of Child Neurology, Department of Pediatrics, Al-Ahli Hospital, Hebron, West Bank, Palestine; c Department of Pediatrics, Princess Alia Hebron Governmental Hospital, Hebron, West Bank, Palestine; d Department of Molecular Genetics & Genetic Toxicology, Arab American University, Ramallah, West Bank, Palestine

**Keywords:** GLRB, hyperekplexia, seizure, startle reflex, case series

## Abstract

**Introduction:**

Hereditary hyperekplexia (HKPX) is a rare neurogenetic disorder caused by mutations in glycine signaling genes, such as GLRB. We report two neonates with autosomal recessive HKPX2 due to a novel GLRB mutation (c.1414C>T, p.Arg472*).

**Cases description:**

Case 1, a 1-month-old female, presented with severe startle responses and tonic episodes, normal EEG, and no developmental delays. Case 2, a 2-week-old male, showed similar symptoms but with generalized rhythmic ictal fast activity on EEG. Both had consanguineous parents and unremarkable brain MRIs. Whole exome sequencing identified the same homozygous GLRB mutation in both cases. Treatment with Clonazepam and Levetiracetam significantly improved symptoms.

**Discussion:**

This is the first report of the c.1414C>T (p.Arg472*) mutation in GLRB, expanding the genetic spectrum of HKPX2. The differing EEG findings highlight the disorder’s phenotypic variability. Early diagnosis and treatment are crucial to prevent complications like hypoxia and SIDS.

**Conclusion:**

This article reports a novel GLRB mutation in two neonates with autosomal recessive HKPX2, presenting with divergent EEG findings.

**HIGHLIGHTS**

- First report of the novel GLRB (c.1414c>t (p.arg472)*) mutation in neonatal hereditary Hyperekplexia 2 (HKPX2).
- Two affected neonates displayed divergent EEG findings despite sharing the same mutation.
- Clonazepam and Levetiracetam effectively reduced startle episodes and muscle hypertonia.
- Findings expand the genotypic and phenotypic spectrum of hereditary HKPX.

## Introduction

Hereditary hyperekplexia (HKPX) is a rare neurogenetic disorder characterized by exaggerated startle responses and persistent muscle stiffness, stemming from dysfunction in glycinergic inhibitory neurotransmission. Mutations in genes involved in glycine signaling within the central nervous system, such as GLRA1, GLRB, and SLC6A5, disrupt inhibitory control, leading to hyperexcitable reflex circuits and impaired motor regulation^[1,2]^. HKPX is classified into four subtypes (HKPX1–4) based on the specific gene mutation and mode of inheritance, with autosomal recessive mutations in GLRA1 being the most common^[1,2]^. Very few cases of all HKPX types have been documented worldwide^[3]^, highlighting its rarity and the need for greater awareness among clinicians.

Neonates with HKPX typically present with hallmark clinical features, including axial hypertonia, exaggerated startle responses to auditory or tactile stimuli, and episodic apnea. These symptoms pose significant risks, including hypoxia and sudden infant death syndrome (SIDS). Unlike secondary causes of hypertonia or startle syndromes, such as pontine lesions or autoimmune conditions, hereditary HKPX is characterized by normal findings on brain imaging, metabolic studies, and routine laboratory tests^[4,5]^.

Electroencephalogram (EEG) studies are also typically unremarkable in HKPX, distinguishing it from epileptic or structural causes of neonatal hyperexcitability^[4,5]^. Despite this, the disorder can result in persistent developmental challenges, highlighting the importance of timely intervention^[5]^. Management of HKPX is critical for managing acute episodes and preventing complications like hypoxia^[4]^. Although HKPX symptoms improve with age, some patients experience lifelong startle responses and movement difficulties. A minority develop phobic anxiety or gait instability, affecting mobility and quality of life^[6]^.

This case series presents two neonates with autosomal recessive HKPX2 caused by a novel GLRB mutation, each displaying distinct EEG findings.

## Cases description

### Case 1

This report details a case of a 1-month-old female presented to our department with severe startle responses and recurrent tonic episodes. The patient, born with a birth weight of 2.7 kg at term to consanguineous parents, required neonatal intensive care unit (NICU) admission shortly after birth due to respiratory distress managed with oxygen therapy. Hypoxic-ischemic encephalopathy was diagnosed following perinatal hypoxia. Within hours after birth, the physicians observed concerning episodes of generalized tonic movements lasting less than 1 minute. These episodes involved limb stiffening and were frequently triggered by auditory or tactile stimuli. Associated symptoms included occasional bluish discoloration around the lips but no loss of consciousness or postictal states. During subsequent clinical evaluations, nasal tapping consistently provoked exaggerated myoclonic jerks of the limbs, an abnormal startle reflex that failed to habituate with repeated stimuli. Episodes primarily occurred upon waking and reduced with sleep, and the patient’s development showed no significant delays at this stage.

**Figure 1.**
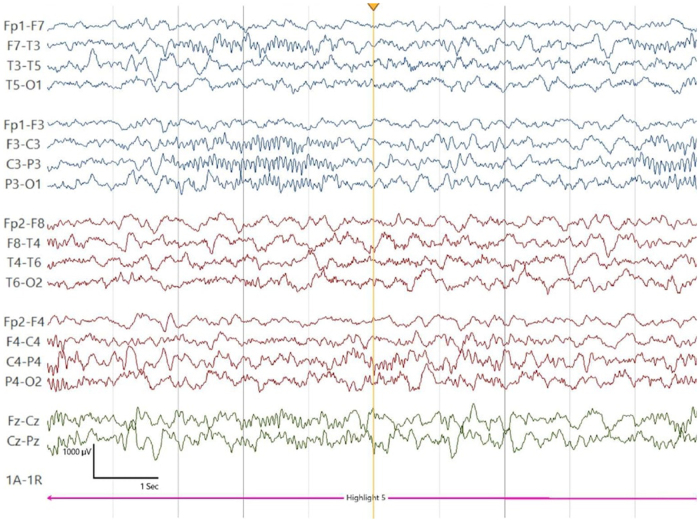
Case 1 19-channel video EEG shows normal interictal background activity, with no evidence of ictal events.

**Figure 2.**
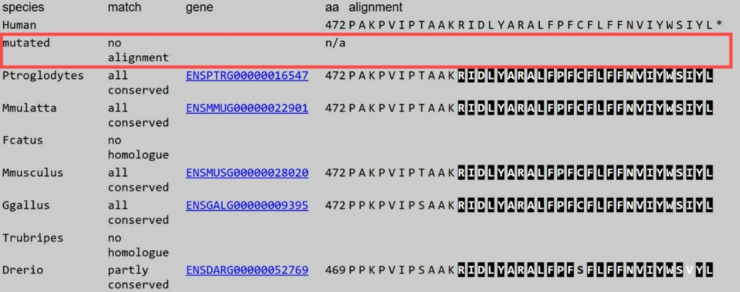
Multiple sequence alignment using the MutationTaster tool demonstrated that this variant leads to the deletion of a conserved region.

Initial investigations revealed normal blood work, and brain magnetic resonance imaging (MRI) was unremarkable, excluding structural abnormalities. However, as shown in Fig. 1, the 19-channel video EEG demonstrated normal interictal background activity. Despite initiating treatment with Phenobarbital (5 mg twice daily), the symptoms persisted. Based on the unusual refractory clinical presentation and the family’s consanguinity, genetic testing was pursued. A provisional diagnosis of HKPX was considered. Whole exome sequencing (WES) test confirmed a pathogenic homozygous mutation in the GLRB gene (c.1414C>T (p.Arg472*)) consistent with the diagnosis of autosomal recessive HKPX2. Additionally, multiple sequence alignment of different regions using MutationTaster tool indicated that the identified variant leads to a deletion of a conserved region (Fig. 2)^[7]^, further supporting the pathogenicity of this mutation.

Treatment with Clonazepam (0.1 mg daily) was initiated, leading to a significant reduction in the frequency of startle responses and tonic episodes. The addition of Levetiracetam (0.5 mL in the morning and 1 mL in the evening) further improved symptoms, particularly muscle stiffness. At the 2-weeks, 1-month and 2-months follow-up, the parents reported a marked improvement, with the episodes decreasing to one every 2 weeks. No feeding difficulties, respiratory issues, or cyanosis were observed. The patient remains under regular monitoring, with plans for comprehensive developmental follow-up to track progress and ensure age-appropriate milestones are achieved. The parents were counseled on the genetic basis of the condition and trained in the Vigevano maneuver (flexion of the head and limbs toward the trunk) to manage acute startle episodes and prevent hypoxia effectively.

### Case 2

A 2-week-old male neonate, born at term with a birth weight of 2.4 kg to consanguineous parents, presented with exaggerated startle responses and generalized muscle contractures. Unlike Case 1, the pregnancy was notable for increased fetal movements, and the delivery was uncomplicated. Postnatally, the infant demonstrated frequent startle responses triggered by auditory and tactile stimuli, accompanied by generalized tonic movements lasting less than one minute. These episodes, similar to Case 1, were not associated with cyanosis, feeding difficulties, or altered consciousness. Physical examination revealed coarse facial features, generalized body rigidity, and exaggerated startle responses involving the head and limbs, which were not observed in Case 1.

**Figure 3.**
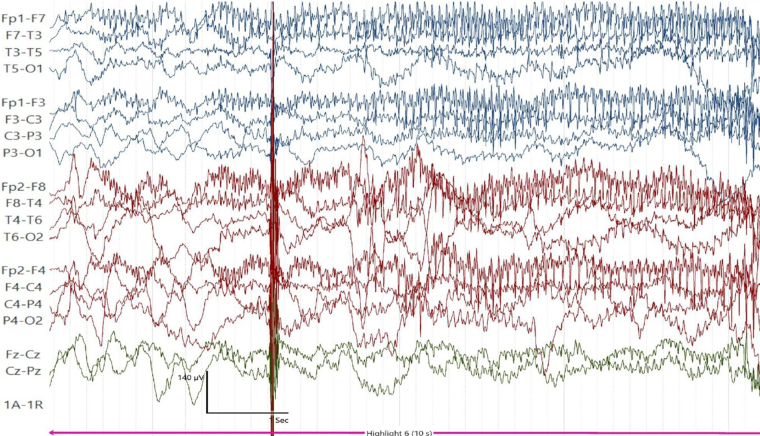
Case 2 19-channel video EEG shows generalized rhythmic nonepileptic fast activity (ictal), clinically synchronized with startle responses, was observed on EEG.

Investigations, including brain MRI, were unremarkable, mirroring findings in Case 1. However, the 19-channel video EEG demonstrated generalized rhythmic ictal fast activity, synchronized clinically with startle responses (Fig. 3), differing from the normal interictal background activity seen in Case 1. Genetic testing via WES identified the same pathogenic homozygous mutation in the GLRB gene (c.1414C>T (p.Arg472*)), confirming autosomal recessive HKPX2.

Treatment with Clonazepam (0.1 mg daily) and Levetiracetam (20 mg/kg/day) led to significant symptom improvement, consistent with the response observed in Case 1. At follow-up, the parents reported satisfactory control of symptoms, with no respiratory or feeding difficulties. The patient remains under regular neurological and developmental monitoring. Genetic counseling and training in the Vigevano maneuver were provided, as in Case 1.

## Discussion

This article highlights a rare presentation of two neonates with an autosomal recessive HKPX2 due to a novel GLRB mutation (c.1414C>T (p.Arg472*)), expanding the genetic and phenotypic spectrum of this rare disorder. While both patients demonstrated classical features of HKPX, their EEG findings diverged significantly. Case 1 demonstrated normal interictal activity, whereas Case 2 showed generalized rhythmic ictal fast activity, synchronized with startle responses. The clinical presentation, diagnostic findings, and treatment response of both cases are summarized in Table 1. HKPX is a rare neurogenetic disorder characterized by exaggerated startle reflexes and sustained muscle hypertonia, often triggered by auditory or tactile stimuli. In neonates, clinical hallmarks include persistent axial stiffness and rigidity that may remain unchanged during positional changes, alongside hyperexcitability evident during startling episodes. These symptoms demand thorough neurological and genetic evaluation to confirm the diagnosis and exclude secondary conditions such as pontine lesions or autoimmune syndromes involving anti-glycine receptor (GlyR) antibodies^[8,9]^.

**Table 1:** Comparative clinical and genetic features of two neonates with HKPX2

Feature	Case 1	Case 2
Age	1 month	2 weeks
Sex	Female	Male
Birth weight	2.7 kg	2.4 kg
Gestational history	Unremarkable	Increased fetal movements
Perinatal course	NICU admission for respiratory distress due to perinatal hypoxia	Unremarkable
Family history	Consanguineous parents, no known neurodevelopmental disorders	Consanguineous parents, no known neurodevelopmental disorders
Presenting symptoms	Severe startle responses, tonic episodes	Severe startle responses, muscle contractures
Triggering stimuli	Auditory and tactile	Auditory and tactile
Episode duration	<1 minute	<1 minute
Associated symptoms	Occasional perioral cyanosis	None
Physical examination	Exaggerated myoclonic jerks, no facial anomalies	Coarse facial features, generalized rigidity
Developmental Status	Normal for age	Normal for age
EEG findings	Normal	Generalized rhythmic ictal fast activity
Brain MRI	Unremarkable	Unremarkable
Genetic mutation	Homozygous *GLRB* (c.1414C>T, p.Arg472*)	Homozygous *GLRB* (c.1414C>T, p.Arg472*)
Treatment	Clonazepam, Levetiracetam	Clonazepam, Levetiracetam
Follow-up	Satisfactory symptom control, no complications	Satisfactory symptom control, no complications
Parental counseling	Genetic counseling provided, trained in Vigevano maneuver	Genetic counseling provided, trained in Vigevano maneuver

This table summarizes the clinical presentation, diagnostic findings, and management of two neonates diagnosed with autosomal recessive HKPX2 due to a homozygous GLRB mutation.

Both patients in this case series displayed the classical features of HKPX, including recurrent exaggerated startle responses, generalized tonic stiffness, and axial rigidity. Importantly, these episodes were not associated with signs of seizures, such as cyanosis, postictal states, or feeding disturbances. While most HKPX cases are associated with normal EEG findings, one patient demonstrated an unusual pattern of generalized rhythmic ictal fast activity, synchronized with startle responses, reflecting nervous system hyperexcitability. This divergence from the typical EEG pattern in HKPX highlights the clinical variability of the condition.

The differential diagnosis of HKPX includes conditions with exaggerated startle responses or muscle stiffness, such as symptomatic HKPX, spasticity, and epilepsy linked to perinatal brain injury or metabolic disorders. Complex genetic neurodevelopmental disorders and acquired causes like brainstem dysfunction, infections (e.g., tetanus), autoimmune conditions, and toxins may also mimic HKPX^[6]^. However, our diagnosis was based on clinical features, MRI, EEG findings, and laboratory tests, ultimately confirmed by genetic testing, ensuring accurate differentiation from these mimicking conditions.

The pathophysiology of HKPX involves defects in glycinergic neurotransmission, an inhibitory mechanism crucial for motor control and the regulation of startle reflexes. Mutations in genes encoding components of the glycine receptor complex – most commonly GLRA1 and GLRB, and less frequently SLC6A5 – impair chloride ion conductance, leading to heightened excitatory responses^[8,10]^. In both cases, the identified GLRB mutation (c.1414C>T (p.Arg472*)) produces a premature stop codon at position 472, truncating the glycine receptor β-subunit and disrupting the last 26 amino acids, a region critical for receptor function. Premature stop codons are typically deleterious and often lead to protein dysfunction^[11]^. This mutation likely reduces cell-surface expression of the glycine receptor, as reported in a prior study of a related mutation in a Jordanian patient^[12]^. This disruption of glycinergic signaling supports the pathogenicity of the variant and its role in the phenotypic manifestations observed in both patients.

To our knowledge, this is the first published article describing this mutation variant and the clinical phenotype associated with it. While this variant has been submitted to ClinVar (3 submissions), no articles currently exist documenting its pathogenicity or associated phenotype. However, some previous studies have described other pathogenic variants in the GLRB gene^[5,13]^. Notably, this mutation affects the same codon as the previously reported c.G1415A (p.R450X) variant, identified in a Jordanian patient with neonatal HKPX[12]. Both mutations disrupt the final transmembrane domain (TM4) of the GlyR β-subunit, which is critical for receptor assembly and membrane anchoring. Functional studies on p.R450X have demonstrated reduced cell-surface expression and GlyR dysfunction, strongly supporting the pathogenicity of premature stop codons at this site^[12]^. A case similar to this report involved a male neonate from a consanguineous marriage who presented with exaggerated startle responses, muscle stiffness, and respiratory distress. Despite multiple antiseizure treatments, a significant improvement was noted with Clonazepam. WES test identified a homozygous mutation (c.97delA) in the GLRB gene, associated with HKPX2, leading to a different amino acid substitution (p.Lys34fs*27)^[14]^. Another reported case of a neonate with exaggerated startle responses and muscle hypertonia was linked to a homozygous mutation in the GLRB gene (p.R212Ter). Unlike our patients, this case involved a distinct variant and demonstrated ongoing neurodevelopmental challenges^[15]^. Functional studies on similar GLRB variants, such as p.Tyr492Cys and p.R450X, suggest that disruptions in this region critically impair glycine receptor function, highlighting the pathogenic significance of this domain^[12]^.

Clonazepam, a benzodiazepine that enhances GABAergic signaling, is the first-line treatment for HKPX^[12]^, with 95% of patients experiencing symptomatic relief^[16]^. In our case, its use resulted in a marked reduction in startle responses and hypertonia. Levetiracetam was added to address muscle stiffness and improve energy metabolism, which further ameliorated symptoms. Caregiver education on techniques such as the Vigevano maneuver was essential in preventing complications like hypoxia during startle episodes^[17]^. Further, genetic counseling is advised for families with a known mutation to guide future reproductive decisions. Despite effective symptom management, HKPX carries risks such as SIDS, particularly in cases involving apneic episodes^[18]^. Our cases add to this body of evidence by demonstrating the efficacy of Clonazepam and Levetiracetam in managing symptoms. While both patients responded well to Clonazepam and Levetiracetam, long-term follow-up is essential to monitor for potential neurodevelopmental delays or complications, which have been reported in other cases of HKPX.

Although HKPX is a treatable condition, it can lead to developmental and neuropsychiatric complications. Intellectual disability was reported in 43.75% of patients, while delayed gross motor development and speech delay were observed in 25% and 18.75%, respectively^[5]^. These findings suggest that the condition’s impact extends beyond motor symptoms, affecting broader aspects of neurodevelopment.

This case series highlights the potential for atypical EEG findings in HKPX, challenging the prevailing notion that EEG remains unremarkable in all cases. Clinicians should consider EEG variability when evaluating neonatal hyperexcitability. The identification of a novel GLRB mutation broadens our understanding of the genetic landscape of this rare condition and highlights the variability in its clinical and electrophysiological presentations. Further research is needed to elucidate the genotype-phenotype correlations in HKPX and to explore the long-term outcomes of patients with novel GLRB mutations.

This case series has been reported in line with the process guideline^[19]^.

## Conclusion

This article described a novel GLRB mutation in two neonates diagnosed with autosomal recessive HKPX2. Both showed divergent EEG patterns, highlighting the phenotypic variability of the disorder. Early initiation of Clonazepam and Levetiracetam resulted in effective symptom control.

## Data Availability

Not applicable.
